# Short-coupled ventricular ectopics leading to cardiac arrest in a young woman

**DOI:** 10.1186/s43044-022-00272-y

**Published:** 2022-04-25

**Authors:** George Katis, Benedict Wiles, Magdi M. Saba

**Affiliations:** 1 St. George’s Hospital, London, UK; 2 Advanced Ventricular Arrythmia Training and Research (AVATAR) Program at St. George’s Hospital, London, UK

**Keywords:** Ventricular ectopic (VE), Short-coupled (R-on-T), Ventricular fibrillation (VF) Catheter ablation, Sudden cardiac death, QRS morphology, Implantable cardioverter-defibrillator (ICD)

## Abstract

**Background:**

This case report highlights the importance of recognizing that ventricular ectopy may be a cause for syncope and sudden cardiac death, through triggered disorganized arrhythmia. In the context of syncope, ventricular ectopy should be carefully assessed for coupling interval and morphology.

**Case presentation:**

A 39-year-old woman, who had presented with recurrent syncope, had a cardiac arrest shortly after admission that required emergency defibrillation. Review of her cardiac monitoring revealed an episode of polymorphic ventricular tachycardia which had degenerated into ventricular fibrillation. The dysrhythmia had been initiated by a short-coupled (R-on-T) ventricular ectopic (VE) beat. Anti-arrhythmic therapy was initiated in the form of hydroquinidine, but the patient continued to have frequent VEs of right bundle branch block (RBBB) morphology with a relatively narrow QRS complex and a variation in frontal axis. A cardiac MRI revealed late gadolinium enhancement of the posterior papillary muscle (indicative of focal scarring). The patient underwent electrophysiological mapping and catheter ablation of her ectopy. The patient made a good recovery and was discharged from hospital with a secondary prevention implantable cardioverter-defibrillator (ICD) in situ.

**Conclusions:**

Short-couped VEs that are superimposed onto the preceding T wave (R-on-T) are indicative of electrical instability of the heart and should prompt urgent investigation. By studying the morphologies and axes of the QRS complexes produced by VEs, we can identify their likely origins and ascertain their clinical significance.

## Background

The QRS morphology of ventricular ectopy on a 12-lead ECG helps to identify their anatomical origin, as well as their clinical significance [1]. VE beats most commonly arise from the outflow tracts of both ventricles, with a greater preponderance of right ventricular beats [2]. Classically benign, outflow tract ectopics have an inferior axis (positive QRS in the inferior leads) and a left bundle branch block pattern if of right ventricular outflow tract origin and an atypical RBBB pattern if from the left ventricular outflow tract. There are different patterns to outflow tract arrhythmia; they can present as isolated beats, couplets, non-sustained and sustained monomorphic ventricular tachycardia [1].

Arrhythmias arising from the His-Pukinje conduction system, typically the terminal arborizations of the left bundle branch fascicles, can also cause both ventricular ectopy and sustained ventricular tachycardia. The resulting QRS complexes have a RBBB-like morphology, but may have either a superior or inferior axis, depending on whether they originate from the posterior or anterior fascicles, respectively [1].

Fascicular QRS complexes have a relatively narrow QRS (< 130 ms) with a sharp initial deflection, consistent with utilization of the rapidly-conducting fascicular tissue [1]. A QRS feature that distinguishes an ectopic of fascicular origin from other left ventricular ectopics is the presence of an R prime wave (R’) of greater amplitude than the R wave in leads V1 and V2, as would also be observed in a typical RBBB pattern [3].

Ectopics arising from left ventricular papillary muscles produce a more atypical RBBB appearance [1]. They are more likely to produce slightly variable QRS complexes and would usually have an R wave greater than the R’ wave in V1 [3, 4]. The varying morphology that is exhibited is due to a single intrapapillary focus, most commonly closer to the tip of the muscle, that has different exits towards the base [4].

Sudden arrhythmic death syndrome (SADS) affects more than 500 people in the UK each year [5]. This report describes a patient with a structurally normal heart in whom short-coupled ventricular ectopy led to cardiac arrest. Isolated VEs are an extremely common finding in healthy individuals. However, short-coupled VEs, those occurring less than 400 ms after the preceding QRS, have the potential to trigger life threatening ventricular rhythms [6]. Their presence, especially in a patient with syncope, requires expert evaluation. Catheter ablation is a potentially curative treatment for short-coupled ventricular ectopy.

## Case presentation

**Fig. 1:**
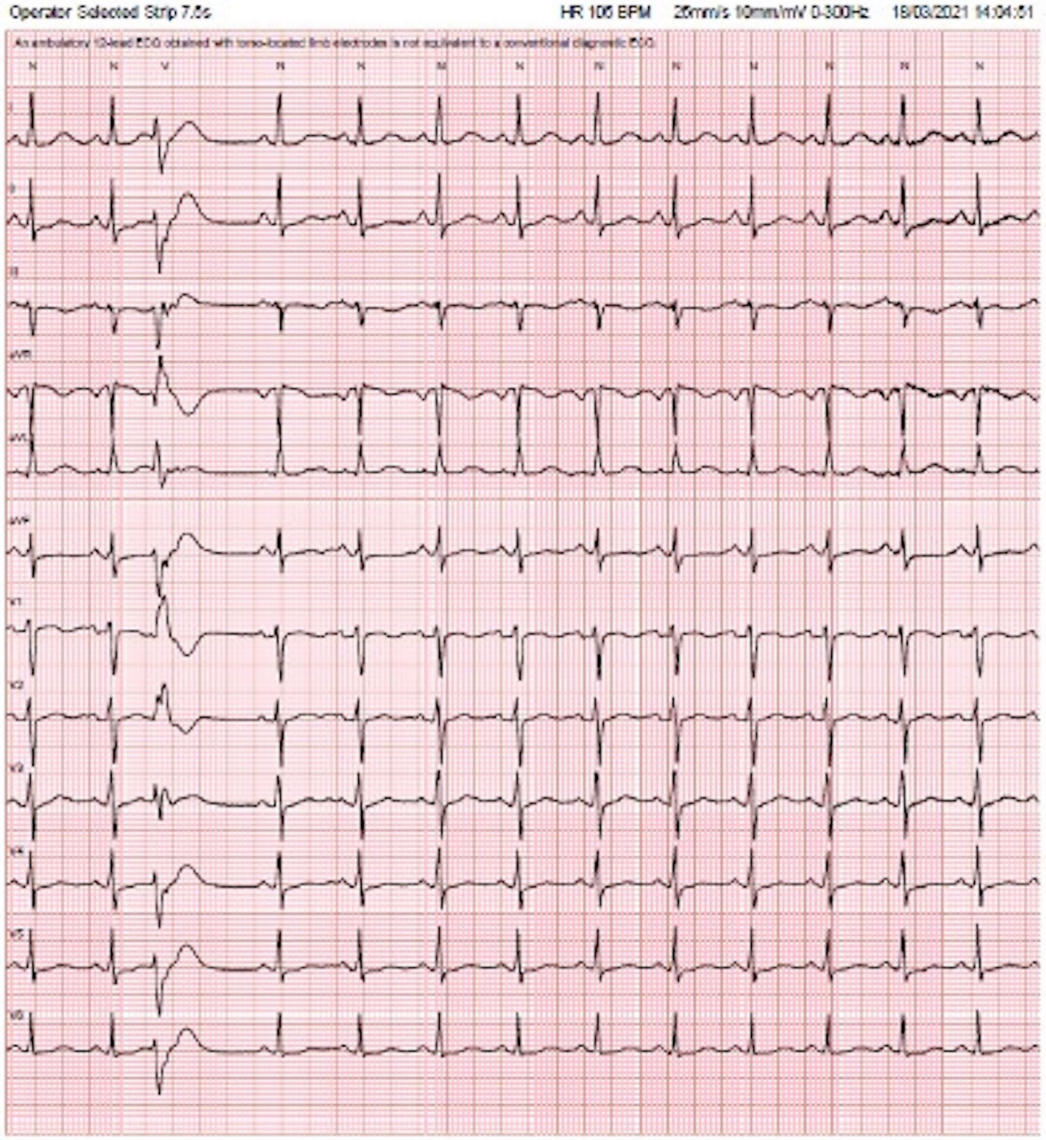
Sinus rhythm with normal QRS complexes and intervals and a single VE of RBBB-right superior axis morphology, demonstrating the r > R′ pattern in V1 and V2 (suggests fascicular origin)

A healthy 39-year-old woman with no significant comorbidities presented with a six month history of recurrent episodes of syncope. Episodes had occurred with no warning and were frequently preceded by palpitations. Physical examination revealed no abnormality and a twelve lead ECG showed sinus rhythm with normal axis, QRS duration and QT interval (Fig. 1).

Shortly after being admitted for further evaluation, she was found to be unresponsive and pulseless on the ward. Cardiopulmonary resuscitation was commenced, with four shocks delivered for ventricular fibrillation, in combination with 300 mg amiodarone and 1 mg adrenaline. Return of spontaneous circulation was achieved after nine minutes and her prior cardiac monitoring was then reviewed.

## Investigations

**Fig. 2:**
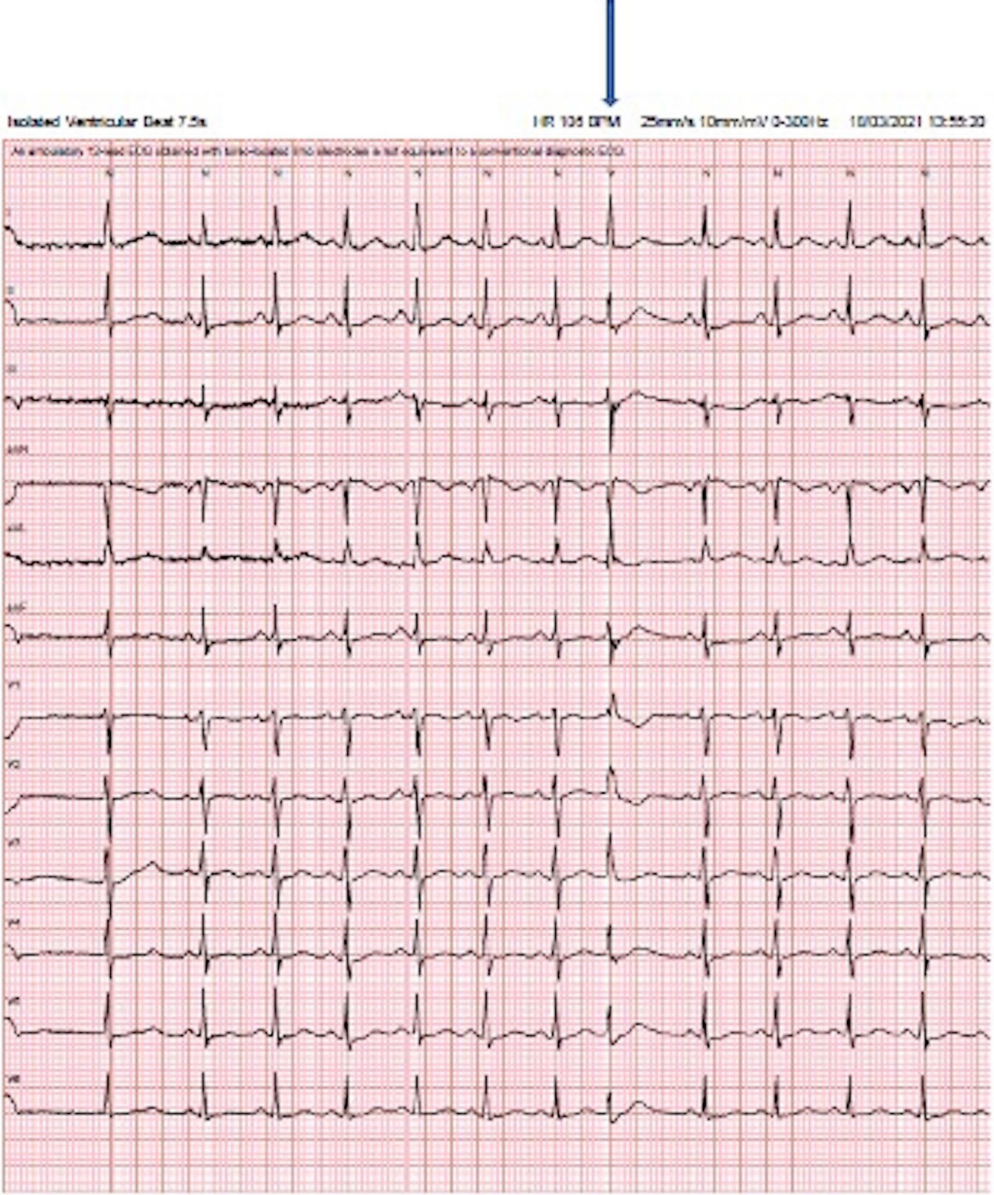
Very narrow QRS in a RBBB pattern with a normal axis. The VE is barely distinguishable from sinus beat in lead 1 (see arrow). The very narrow QRS and the r > R′ pattern in V1 strongly suggest a fascicular origin

**Fig. 3:**
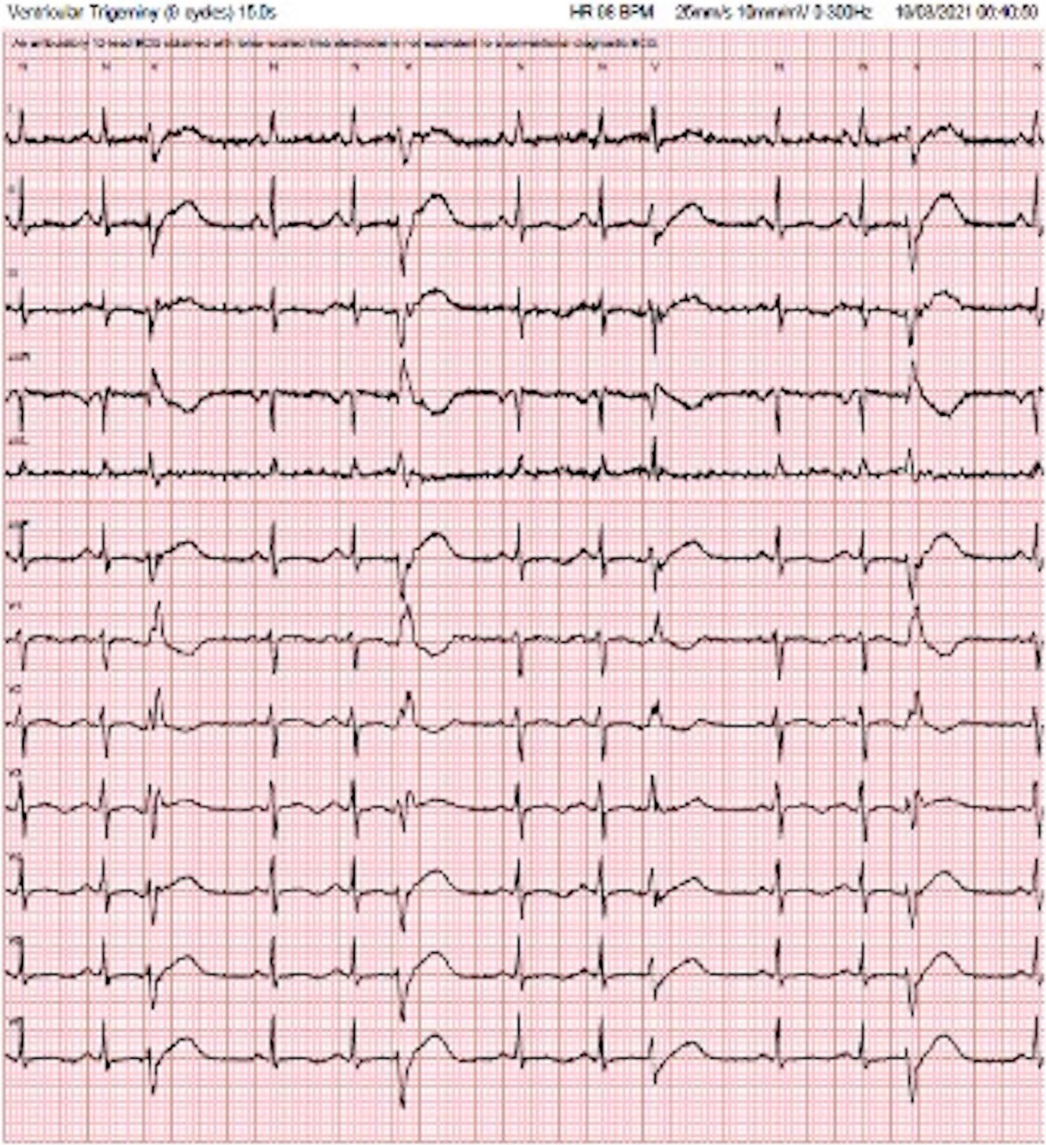
RBBB with wide QRS displaying the atypical variability, seen most clearly in V1 and V2 with differences seen in each ectopic beat (suggests papillary origin)

Ventricular fibrillation was observed to have been initiated by a short-coupled VE resulting in an ‘R-on-T’ phenomena. Post arrest ECGs also demonstrated this with frequent VEs of varying morphology, consistently occurring within 400 ms of the preceding QRS, in which the R wave landed on the upslope of the T wave. The ectopics all had a RBBB-like morphology, indicating a left ventricular origin, with features suggestive of both fascicular and papillary muscle origins (Figs. 1, 2, 3).

An echocardiogram, coronary angiogram, signal-averaged ECG, and high-chest lead ECG (for Brugada syndrome) were all normal. Genetic testing, including a full molecular autopsy panel consisting of 130 genes, was sent for analysis. A cardiac MRI showed normal cardiac structure and function, except for a small area of scarring on the posterior papillary muscle of the left ventricle, highlighted by late gadolinium enhancement.

**Fig. 4:**
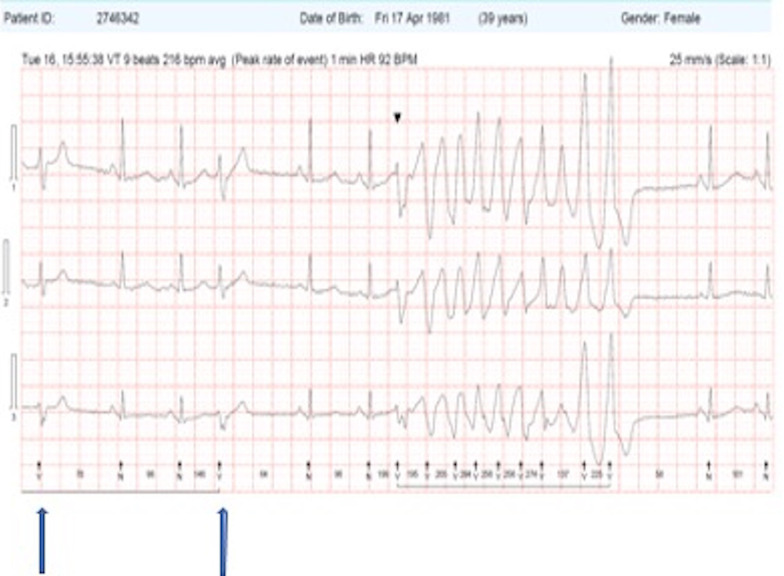
Short-coupled VE (coupling interval 310 ms, on the peak of the T wave) leading to non-sustained polymorphic VT in a 3-lead ECG. Compare to the first two VEs (see arrows) on the same tracing, which are much-later coupled and trigger no such arrhythmia

A 24-h ECG captured further episodes of R-on-T with self-terminating polymorphic VT (Fig. 4) induced. In total, over 20,000 VE beats occurred in a 24-h period, an 18% burden. Despite a reduced ectopic burden after treatment with hydroquinidine, there remained > 4000 VEs of a similar morphology to the offending VE which led to the arrest.

## Treatment

The patient was initiated on a class 1A anti-arrhythmic agent in the form of hydroquinidine. This reduced her ectopic burden but did not result in complete suppression. As the cause of the arrest was short-coupled PVC’s which persisted in the absence of electrolyte imbalance, this is non-reversible and therefore the implantation of an ICD for secondary prevention of sudden cardiac death was indicated. An electrophysiological study was performed with successful mapping and ablation of the ventricular ectopy. These were found to arise from the fascicular insertions of the antero-lateral and posteromedial papillary muscles.

**Fig. 5:**
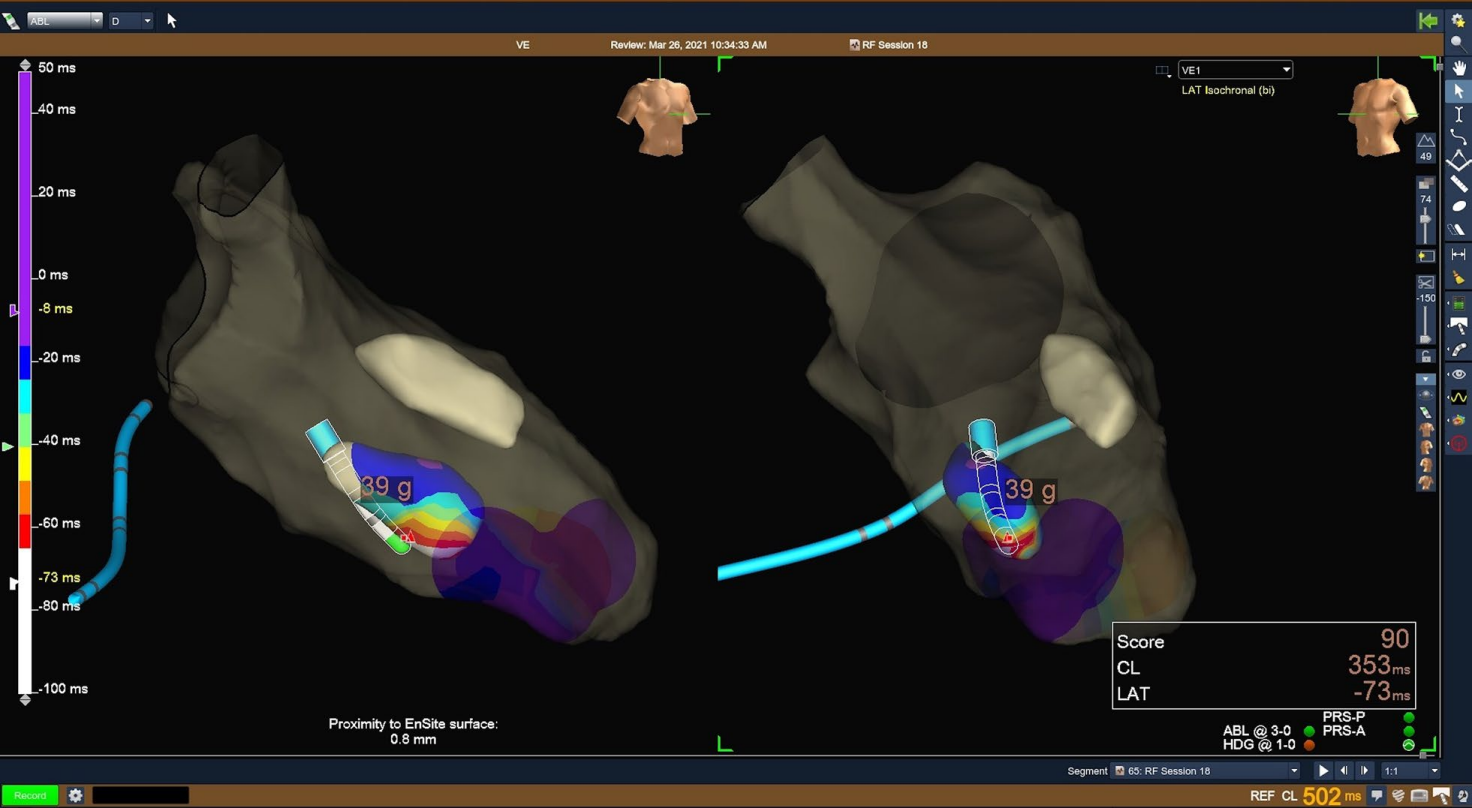
Three-dimensional electro-anatomic map of the left ventricle seen in the right anterior oblique (left) and the caudal (right) projections, illustrating one of several sites of origin of ectopy targeted with ablation. The catheter tip (green) is shown at the site of earliest activation (white and red area) of a frequent VE

Left ventricular mapping was carried out using the Precision 3D electro-anatomical mapping system and the HD Grid mapping catheter through an Agilis deflectable sheath via a trans-Mitral approach (Fig. 5). Trans-oesophageal echocardiogram (TOE) was used to visualize the papillary muscles. Consistent with the ECG findings, activation mapping revealed the site of origin of the ectopy, involving the papillary muscles and the fascicular tissue surrounding them. Once the site of origin of the clinical ectopy was defined using activation mapping, the Tacticath ablation catheter was introduced and radio-frequency energy (40 Watts) was applied to the earliest sites of activation on the papillary muscles and sites of abnormal fascicular electrograms at the bases of both papillary muscles, guided by TOE. On the antero-lateral papillary muscle, ablation induced sustained monomorphic VT, similar in morphology to one of the clinical ectopics, which slowed and terminated after more than 60 s of energy being applied. Subsequently, there were no further episodes of sustained VT during the procedure.

## Outcome and follow-up

On discharge it was advised that all first-degree relatives should undergo cascade screening in a specialized, inherited cardiac conditions clinic. Eleven months after discharge, the patient remains asymptomatic and device interrogation revealed no further episodes of sustained ventricular dysrhythmia.

## Conclusions

Demonstrated in our electrical traces above is an RBBB ectopic often with a narrow QRS (Fig. 2). Indeed, the extremely narrow QRS of these ectopy provide a rare finding in this case because they are very difficult to discern from an atrial ectopic or sinus beat, meaning their significance could easily be missed, leading to misdiagnosis. Along with the typical r < R′ pattern, these features favor a fascicular origin. However, the variable axes, often broad QRS and the atypical variable morphologies strongly suggest localization to the papillary muscles. These interesting alternating morphologies resulted in the culprit tissue being mapped to the fascicular insertions in both papillary muscles. In anatomical studies, Purkinje fibers have been shown to drape over both apical and basal parts of the papillary muscles [7]. However, activation appears to be localized to specific junctions at the base [7]. This provides an important link between the fascicular tissue and papillary muscle, as reentrant circuits of fascicular VT may involve the Purkinje fibers surrounding the papillary muscles [8]. The ectopy described in this case has features consistent with both fascicular and papillary muscle origins.

Regardless of QRS morphology, short-coupled VEs that are superimposed on the preceding T wave (R-on-T) should prompt urgent investigation as they are a warning sign for electrical instability of the heart muscle [1]. One important caveat to this case is that there remains no exact definition of short-coupled VEs as an independent diagnosis [9], which creates uncertainty when reporting findings and comparing the incidences recorded. In a recent study, Almehairi et al. [10] propose a unifying diagnosis for such arrythmias which would differentiate them from the umbrella term of “idiopathic VF”—namely “Idiopathic short-coupled ventricular tachyarrhythmias”.

## Data Availability

Detailed in the reference list.
